# A new method for the inoculation of *Phytophthora palmivora* (Butler) into cacao seedlings under greenhouse conditions

**DOI:** 10.1186/s13007-020-00656-8

**Published:** 2020-08-19

**Authors:** Paola Delgadillo-Durán, Mauricio Soto-Suárez, Leonora Rodriguez-Polanco, Martha Carrero-Gutierrez, Esperanza Torres-Rojas, Roxana Yockteng

**Affiliations:** 1 Corporación Colombiana de Investigación Agropecuaria (AGROSAVIA), Centro de Investigación Tibaitatá, Km 14 vía Mosquera, Bogotá, Colombia; 2 Corporación Colombiana de Investigación Agropecuaria (AGROSAVIA), Centro de Investigación Nataima, Km 9 vía Espinal-Chicoral, Tolima, Colombia; 3 Universidad Nacional de Colombia, Bogotá, D.C Colombia; 4 Institut de Systématique, Evolution, Biodiversité-UMR-CNRS 7205, National Museum of Natural History, Paris, France

**Keywords:** Black pod, *Theobroma cacao*, Image analysis, Inoculation method, Resistance evaluation

## Abstract

**Background:**

The black pod disease affects cacao plantations worldwide; it is caused by the oomycete species of the genus *Phytophthora*. The resistance of cacao plants to the black pod is commonly evaluated by artificial inoculation of the pathogen and the monitoring of the disease symptoms. However, it is difficult to identify resistant plants because the commonly used methods for the inoculation of the pathogens produce inconsistent results. Therefore, this study aimed to develop an efficient and reliable method to evaluate the resistance of *Theobroma cacao* seedlings to the infection by *Phytophthora palmivora.*

**Results:**

Seedlings of different cacao genotypes were inoculated with *P. palmivora* under greenhouse conditions using the previously reported inoculation methods and a newly proposed method, the agar–water solution method. While none of the previously reported methods was effective, the agar–water solution method ensured a 100% seedling infection under greenhouse conditions. The proposed agar–water methodology is fast, simple and reproducible. Furthermore, the evaluation of this method in susceptible (CCN-51) and tolerant (SCA-6) *T. cacao* genotypes produced the expected contrasting results.

**Conclusions:**

The agar–water solution method presented in this study is an efficient alternative inoculation protocol for the identification of cacao genotypes that are resistant to black pod under greenhouse conditions.

## Background

*Phytophthora* (Mont.) de Bary is a large genus of the oomycete class with more than 80 species [1] that attacks cultivated plant species [2, 3]. This genus shares several biochemical and structural features with plants and algae. These include, mitochondrial ridges, the presence of β-1,3 and β-1,6 glucans and the absence of chitin in the cell wall, and the synthesis of lysine via diaminopimelic acid [4]. In cacao (*Theobroma cacao* L.), the incidence and severity of the disease caused by *Phytophthora* spp. depend on local environmental conditions [5–7]. The annual losses from black pod, the common name of the cacao disease caused by several *Phytophthora* spp., have been estimated to be as much as 30% of total crop production [8]. This translates into annual losses of approximately 3800 million US dollars for producers around the world [1]. When the conditions are suitable for the pathogen, cacao plantations can be completely devastated [9].

Although several *Phytophthora* species can infect cacao plants, only a few are considered important because of the extensive damage that they cause. In cacao plants, black pod is caused by four species of the genus *Phytophthora*: *P. palmivora*, *P. megakarya*, *P. citrophthora* and *P. capsici*/*P. tropicalis* [1]. *Phytophthora palmivora* (Butler) infects more than 200 crops globally, including potato, durian, coconut, pineapple, rubber, citrus, papaya, oil palm and cacao [10–12]. It is a cosmopolitan species occurring in all cacao-producing countries and one of the most destructive *Phytophthora* species [11, 13]. The rapid decomposition of the infected tissues by *P. palmivora* is the main limitation in seedling production in tropical regions [14, 15], and in cacao bean production and its quality [16].

The identification of materials with genetic resistance to pathogens is a promising alternative for disease control [9, 17, 18]. Qualitative and quantitative evaluations of phenotypic resistance under laboratory, greenhouse or field conditions are now possible. The evaluations can combine the traditional methods and high-performance technologies, such as non-invasive hyperspectral images, robotics or remote sensing [19]. However, the current protocols for the evaluation of resistance to *P. palmivora* in the field and in vitro are based mainly on the observation of the infection after the artificial inoculation of detached leaves or pods [9, 20, 21]. Inoculation is usually done by using fragments of infected tissue, agar discs or mycelium suspensions [1, 22, 23], and this has not always produced consistent and reliable results in establishing the resistance of the cacao genotypes [24, 25]. In addition, the use of detached leaves does not allow for differentiating between the plant response to the stress caused by cutting the leaves and the response to the infection caused by the pathogen.

This study presents an efficient method for the inoculation of *P. palmivora* in *T. cacao* seedlings. It facilitates the identification of the extent of resistance at the initial stages of plant development. This method is valuable for breeding programs because it provides a fast and reproducible approach to screening for *P. palmivora* resistance in germplasm collections.

## Results

### Initial evaluation of the inoculation methods

Two susceptible genotypes of *T. cacao*, IMC-67 and CCN-51 were selected to evaluate the infection caused by *P. palmivora* using various inoculation techniques. Direct inoculation into the soil, inoculation by spraying the foliar area and inoculation by applying agar discs directly on the cacao leaves were tested [22, 23, 26] and compared to the proposed agar–water method. To determine the appropriate inoculum doses, several concentrations of the highly virulent *P. palmivora* Tocha-325 strain were tested (3 × 10^5^ to 1 × 10^7^ zoospores/ml).

Under the evaluated conditions, none of the previous methods effectively infected the cacao seedlings (i.e. indications of early infection such as chlorosis and necrosis) (Additional file 1). The spraying and soil inoculation protocols were not effective as no symptoms were observed after 10 days of the initial inoculation.

The agar disc protocol produced lesions in the six inoculated plants of the IMC-67 genotype after 24 h; these lesions were localized on the area where the agar was added, and after 96 h no progression to the other tissues was observed. In contrast, the agar–water solution method infected 100% of the evaluated plants of IMC-67 (10 in total) and the symptoms were observed 48 h after inoculation for both concentrations, 1 × 10^7^ and 1 × 10^8^ zoospores/ml. All the infected seedlings of the IMC-67 genotype exhibited brown necrotic lesions and chlorosis and no symptoms were observed in the negative controls.

### Validation of the agar–water method in the susceptible genotypes

The symptoms of infection produced by this protocol were confirmed using CCN-51, a cacao genotype that is known to be very susceptible to *Phytophthora* sp. The comparison of the inoculation by the spraying method and the agar–water solution method (1 × 10^7^ zoospores/ml) confirmed the efficiency of the latter. The nine plants inoculated with the pathogen by spraying did not exhibit symptoms after 48 h. In contrast, all the nine inoculated with the agar–water protocol showed visible symptoms (Additional file 1). These results were used to standardise the inoculation protocol described below.

### Description of a standardised inoculation protocol

#### Protocol

The viability of the strain should be verified before the plants are inoculated with the pathogen. A mycelial disc should therefore be placed on the surface of a previously disinfected pod obtained from a susceptible genotype. The pod should be wrapped in a plastic bag to increase the relative humidity. After 3 or 4 days, the pathogen should be re-isolated in a carrot agar medium. To guarantee successful infection, the seedlings should not be older than 3 months after grafting. The protocol is as follows:

1. Allow oomycete *Phytophthora palmivora* colonies to grow on a carrot agar medium (18:100 g) in Petri dishes for 15–17 days at 25 °C.
2. Perform thermal shock on 17-day-old pathogen cultures in each Petri dish to release the zoospores in accordance with the methodology of Lawrence, 1978 [27], modified by Phillips-Mora, 1989 [28].
3. To each Petri dish, add 15 ml of cold distilled water (± 4 °C), and homogenise the solution with a sterile brush.
4. Incubate the Petri dishes for 30 min in a dark chamber at 4 °C.
5. Incubate the Petri dishes for 30 min at 25 °C and count the zoospores by using a Neubauer chamber.
6. Prepare 7 ml of 0.4% (w/v) agar–water medium.
7. Add the agar–water prepared in Step 6 to the petri dish with zoospores, and adjust the zoospore concentration to 1 × 10^7^ zoospores/ml.
8. Homogenise the mixture with a sterile swab until the mixture has a gel like structure; then proceed to the next step.
9. With a new sterile swab, place approximately 1 cm^3^ of the homogenized mixture of the zoospore on the center of the abaxial side of adult leaves. Use a new swab to inoculate each plant.
10. Place wet paper towels at the base of the stem to maintain the relative humidity.
11. Finally, wrap the plant in a transparent plastic bag to maintain the moisture.

The first symptoms, small necrotic spots with a thickness of 1 mm, are expected to appear after 24 h of inoculation. As the infection progresses, the necrotic lesions and chlorosis become more evident. It is important to mention that the timing of the appearance of the first symptoms of infection might vary by genotype.

### Protocol validation and phenotypic analysis

**Fig. 1:**
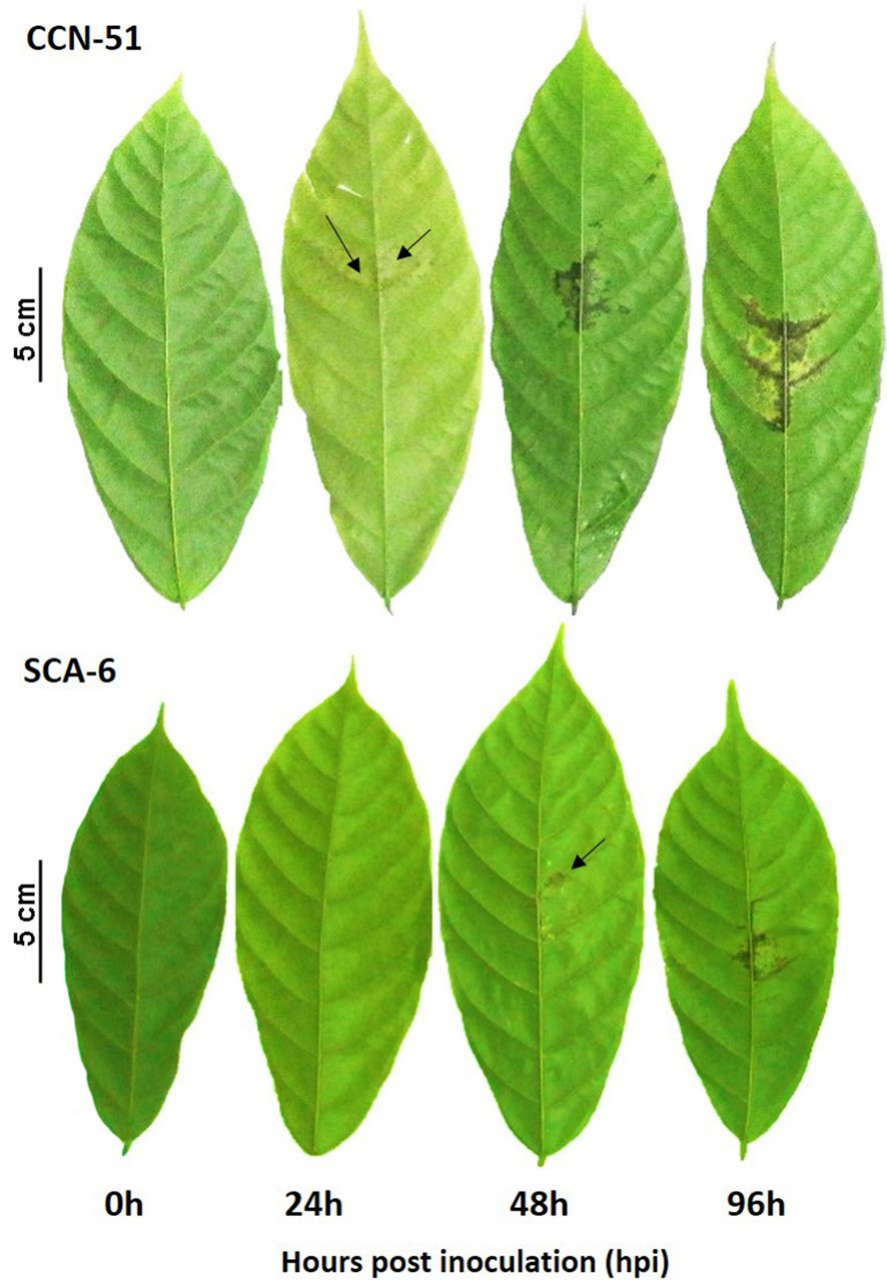
Symptoms of the disease from 0 to 96 hpi in the susceptible CCN-51 genotype and in the tolerant SCA-6 genotype infected with *Phytophthora palmivora* using agar-water solution

**Fig. 2:**
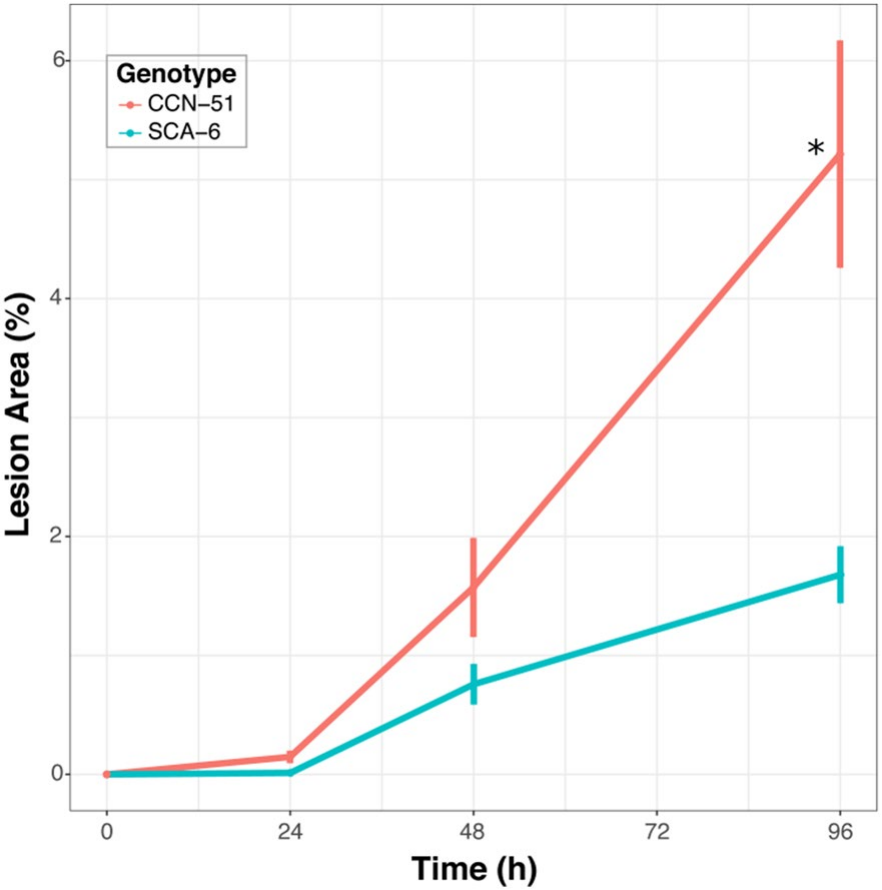
Progress of the lesion area in the two genotypes. Each point represents the mean of the percentage of the affected area at different time points of the three biological replicates (n = 9 plants per hour for each genotype). (*) This indicates the statistically significant difference between the susceptible and tolerant genotypes, as determined by the analysis of variance. The error bars represent the standard deviation

The agar–water protocol was validated with cacao seedlings of the CCN-51 (susceptible) and SCA-6 (tolerant) genotypes. The symptoms were evaluated in a total of 36 plants per genotype (9 per hpi) at 0, 24, 48 and 96 h post inoculation (hpi). All the plants exhibited symptoms of infection at 96 hpi (Additional file 2), but the responses of the susceptible and tolerant genotypes were different. At 24 hpi, small necrotic spots appeared in the susceptible plants of the CCN-51 genotype (Fig. 1). In contrast, no symptoms were visible in the tolerant plants of the SCA-6 genotype at 24 hpi. After 48 hpi, the two genotypes manifested symptoms, and the lesion area increased considerably in both at 96 h (Figs. 1 and 2). As was expected, the highest percentage of lesion area was observed in the susceptible plants (Figs. 1 and 2). Statistical evaluations were performed using an analysis of variance (ANOVA; *p* ≤ 0.05) and a Tukey’s test (Additional file 3). The mean percentage of the lesion area at 96 hpi in the susceptible plants was 5.2%, which was significantly higher than that in the tolerant plants (1.68%) (Fig. 2, Additional file 3).

## Discussion

The evaluation of the resistance of cacao genotypes against *Phytophthora palmivora* has been traditionally conducted with leaf discs or detached pods [13, 20]. These methods trigger plant responses to wounding stress hindering the distinction of genes expressed as part of the disease response (1). The agar–water method does not suffer from this because unwounded leaves from undamaged plants are used. In the present study, the agar–water method consistently infected the cacao seedlings and was more effective than the previously described methods.

### Protocol validation and phenotypic analysis

The agar–water protocol for seedlings exhibited a high efficiency of infection in the IMC-67, CCN-51 and SCA-6 genotypes. All the plants developed lesions at 48 h after inoculation. For the more susceptible genotypes, the CCN-51 and the IMC-67, the symptoms were chlorosis and brown-coloured necrotic lesions, which are the expected symptoms of a *Phytophthora* spp. infection. In contrast, the SCA-6 exhibited smaller lesions after 48 h. Under natural conditions, black pod occurs more frequently in humid conditions. The presence of free water allows zoospores to swim and move towards (chemotactically and electrostatically) tissues where the infection can further develop [29]. The agar–water solution provides a humid environment allowing zoospores to move easily and stay alive until they can infect the plant.

Standardize scales for measuring the progression of the disease in cacao seedlings are not yet available but visual categorization exists for assays with leaf discs and detached pods [20]. Because the symptoms on the seedlings are comparable to those observed on leaf discs, we used the scale from Nyassé et al. [20] in this study. In this scale, the number 0 corresponds to asymptomatic plants, 1 corresponds to very small localised penetration points, 2 corresponds to small penetration points that form a network, 3 corresponds to coalescing intermediate-sized lesions, 4 corresponds to large brown coalescent patches, and 5 corresponds to large uniform dark brown lesions. The lesions observed in the susceptible plants (CCN-51 genotype) after 24 hpi correspond to a stage of 1, and those observed at 96 hpi correspond to stages 4 and 5. In contrast, for the tolerant plants (SCA-6 genotype), the lesions observed after 48 hpi correspond to a stage of 2 and after 96 hpi to a stage of 3.

The analysed genotypes exhibited the expected contrasting behaviour. After 48 hpi, the lesion areas in the two genotypes increased considerably; however, the highest percentage of lesions was observed in the susceptible plant CCN-51 (Additional file 2 and Fig. 2). The results of the ANOVA indicated that the difference was significant (*p* ≤ 0.05; Additional file 3 and Fig. 2). The variations in the appearance of the symptoms observed in the SCA-6 confirmed the findings of previous studies [30]. This genotype has always exhibited tolerance; however, the tolerance response depends on the experimental conditions and the *P. palmivora* strain. The results confirm those of previous studies in which the defence response was dependent on the genotype rather than the analysed inoculation method or plant organ under analysis [20, 24, 31].

## Conclusions

In this study, we present a standardized inoculation protocol for infecting *Theobroma cacao* seedlings with *P. palmivora.* The protocol is simple, efficient, reproducible, and generates a differential response, between susceptible and resistant cacao genotypes. The protocol employed a medium based in an agar–water suspension that maintained the humidity of the zoospores, thus ensuring the penetration of the pathogen in the plant. This protocol was tested on three genotypes, where the disease symptoms appeared at 48 hpi. Image analysis confirmed contrasting responses to *P. palmivora* in the susceptible CCN-51 genotype and the tolerant SCA-6 genotype under nursery conditions. This protocol could be used to evaluate the resistance of cacao seedlings in a breeding program and to study the genes involved in the response to infection by *P. palmivora.*

## Methods

### Plant material

The first inoculation trials were performed by using the reported *T. cacao* CCN-51 genotype, which is susceptible to *Phytophthora*, and the IMC-67 genotype, which is moderately susceptible to this pathogen [13]. Three-month-old seedlings of the CCN-51 and IMC-67 genotypes were used in the assays.

To confirm the efficiency of the selected protocol, the assay was repeated in 3-month-old seedlings of the CCN-51 genotype.

Once the infection was reproducible, the assay was established using two genotypes with contrasting responses to validate the protocol and to perform a phenotypic analysis. The susceptible genotype CCN-51 and the SCA-6 genotype, which has been reported as tolerant to *Phytophthora* spp. were used. Seedlings of each genotype were grafted on 3-month-old plantlets of the IMC-67 genotype, the most common rootstock in Colombia.

### Pathogen material

Tocha-325, a *P. palmivora* strain that was isolated from infected plants in the Chaparral municipality (03° 35ʹ 311ʺ N, 075° 35ʹ 172ʺ W) in the Colombian department of Tolima, was used. In previous experiments, this strain was found to be more virulent than several other isolated strains (data not shown). The strain was conserved in a V8 agar medium (200 ml of V8 juice with 3 g of CaCO_3_, and 13 g of agar–agar dissolved in 1 l of sterile distilled water). It was then cultured for the assay in a carrot agar medium (100 g of peeled ground carrots and 13 g of agar–agar in 1 l of sterile distilled water).

### Inoculation preparation

The Tocha-325 strain had been previously reactivated in cacao pods of the susceptible CCN-51 genotype. Single colonies were then selected and grown in Petri dishes with carrot agar medium for 15 to 17 days at 25 °C. The zoospore suspension from the solid media was prepared in accordance with the methodology of Lawrence, 1989 [27], as modified by Phillips-Mora and Galindo, 1989 [28].

Next, 15 ml of cold distilled water was added to each Petri dish, and the solution was homogenised using a sterile brush. The solution was kept in a dark chamber at 4 °C for 30 min and subsequently incubated at 25 °C for another 30 min to induce zoospore release. Once the zoospore solution had been prepared, the number of zoospores was counted using a Neubauer chamber (Blaubrand, Germany) and a Primo Star Zeiss microscope (Zeiss, Germany).

### Inoculation tests

The inoculation tests were done at greenhouse conditions with temperature in the range of 30° to 32 °C. In the preliminary tests, inoculation (3 × 10^5^ and 6 × 10^5^ zoospores/ml) was done by direct inoculation into the soil, agar discs and spraying. However, none of these techniques successfully infected the plants of the genotypes CCN-51 and IMC-67 (Additional file 1).

#### Inoculation by agar–water

An agar–water solution at 0.4% (w/v) was prepared with an inoculum as follows: To each Petri dish containing the agar–water solution, 1 ml of the adjusted inoculum solution was added and homogenised and the concentration was adjusted to 1 × 10^7^ and 1 × 10^8^ zoospores/ml. A sterile swab was used to place the inoculum on the abaxial surface of all the leaves of the plants (Additional file 1). As negative control, one plant per biological replicate per treatment was inoculated with the agar–water solution without zoospores. Once inoculated, the plants were placed in a humid chamber for 48 h.

#### Inoculation by agar–water vs inoculation by spraying

The infection efficiency of the inoculation methods involving spraying and applying an agar–water solution was compared. Seedlings of the susceptible CCN-51 genotype were inoculated with the pathogen at a concentration of 1 × 10^7^ zoospores/ml by using an agar–water solution (3 plants) and by spraying the leaf area (10 ml per plant) (3 plants). Each assay was conducted three times for a total of nine plants inoculated by each method. As negative controls, one plant per biological replicate per treatment was inoculated with distilled water. Once inoculated, all the plants were placed in a humid chamber for 48 h.

### Phenotypic analysis

To evaluate the appearance of disease symptoms, all the leaves of 12 seedlings of the susceptible CCN-51 genotype and 12 seedlings of the tolerant SCA-6 genotype were inoculated using the agar–water inoculation protocol. The assay was conducted three times for a total of three biological replicates to maximise the statistical reliability of the data. Each biological replicate was inoculated with 1 day of difference using the same zoospore batch to prepare the inoculum. Within each biological replicate, one plant of each genotype was used as a negative control. To quantify the progress of the disease, three plants of each genotype were photographed at 0, 24, 48 and 96 h after inoculation. All the leaves of each plant were cut at each evaluation time and aligned from youngest to oldest for the photographs. Images were taken with a Canon PowerShot SX500 camera with a resolution of 3000 × 4000 pixels in constant light. Image processing was performed with the Compu Eye, Leaf and Symptom Area software [32]. The lesions in the image were coloured in yellow to be recognised by the program. For the object (the leaf) to be distinguishable from the background, the edge of the leaf was selected to facilitate the classification of the pixels by colour intensity (Additional file 2).

Disease progression was measured as the percentage of the affected area at different time points during the infection process. The percentage of the area in which lesions were observed was evaluated in each seedling. The differences in the phenotypic responses of the two contrasting genotypes were evaluated by one-way ANOVA. In addition, Tukey’s test was performed with InfoStat 2017 software [33] to determine statistical significance of differences between the replicates.

## Supplementary information

**Additional file 1.** Percentage of plants infected in each inoculation assay. The numbers between parentheses represent the number of plants exhibiting symptoms at 48 h of inoculation per the total number of analysed plants.

**Additional file 2.** Images of the leaves from the two genotypes, CCN-51 and SCA-6, at 96 h after inoculation with *Phytophthora palmivora*. The lesion area caused by black pod was coloured in yellow to facilitate the recognition by the Compu Eye LSA software. CCN-51: susceptible genotype; SCA-6: tolerant genotype; TR: technical replicate (three plants per biological replicate).

**Additional file 3.** a. One-way ANOVA of leaf damage caused by *P. palmivora.* b. Tukey’s test for the average of the percentage of the lesion caused by *P. palmivora.*

## Data Availability

The dataset and/or analysed during the current study available from the corresponding author on reasonable request.
