# Supplementary material for: A new method for the inoculation of Phytophthora palmivora (Butler) into cacao seedlings under greenhouse conditions
Source: Plant Methods. 2020 Aug 19;16:114. doi: 10.1186/s13007-020-00656-8 (PMC7437064; doi:10.1186/s13007-020-00656-8)
Supplement: Supplementary file 1 — Additional file 1. Percentage of plants infected in each inoculation assay. The numbers between parentheses represent the number of plants exhibiting symptoms at 48 h of inoculation per the total number of analysed plants. [file 13007_2020_656_MOESM1_ESM.docx]

**Additional file 1.** Percentage of plants infected in each inoculation assay. The numbers between parentheses represent the number of plants exhibiting symptoms at 48 hours of inoculation per total number of analysed plants.

|  |  | **Cacao genotype** | | |
| --- | --- | --- | --- | --- |
|  | **Inoculum concentration (zoospores/ml)** | **IMC-67** | **CCN-51** | **SCA-6** |
| **Inoculation by spraying** | 3x10^5^ | 0 (0/10) | 0 (0/4) | - |
|  | 6x10^5^ | 0 (0/10) | 0 (0/4) | - |
|  | 1x10^7^ | - | 0 (0/9) | - |
| **Inoculation to the soil** | 3x10^5^ | 0 (0/9) | 0 (0/4) | - |
|  | 6x10^5^ | 0 (0/9) | 0 (0/4) | - |
| **Inoculation with agar discs** | 6x10^5^ | 0 (0/6) | - | - |
| **Inoculation with agar-water** | 1x10^7^ | 100 (5/5) | 100 (27/27)* | 100 (18/18) |
|  | 1x10^8^ | 100 (5/5) | - | - |

* The number corresponds to the sum of plants of the genotype CCN-51 used in the two assays, the validation of the method in susceptible genotypes and the phenotypic analysis.
