# Supplementary material for: A new method for the inoculation of Phytophthora palmivora (Butler) into cacao seedlings under greenhouse conditions
Source: Plant Methods. 2020 Aug 19;16:114. doi: 10.1186/s13007-020-00656-8 (PMC7437064; doi:10.1186/s13007-020-00656-8)

**Additional file 2** –Images of the leaves from the two genotypes, CCN-51 and SCA-6, at 96 hours after inoculation with *Phytophthora palmivora*. The lesion area caused by black pod was coloured in yellow to facilitate the recognition by the Compu Eye LSA software. CCN-51: susceptible genotype; SCA-6: tolerant genotype; TR: technical replicate (three plants per biological replicate).

**Biological Replicate 1 - 96 hours post inoculation**


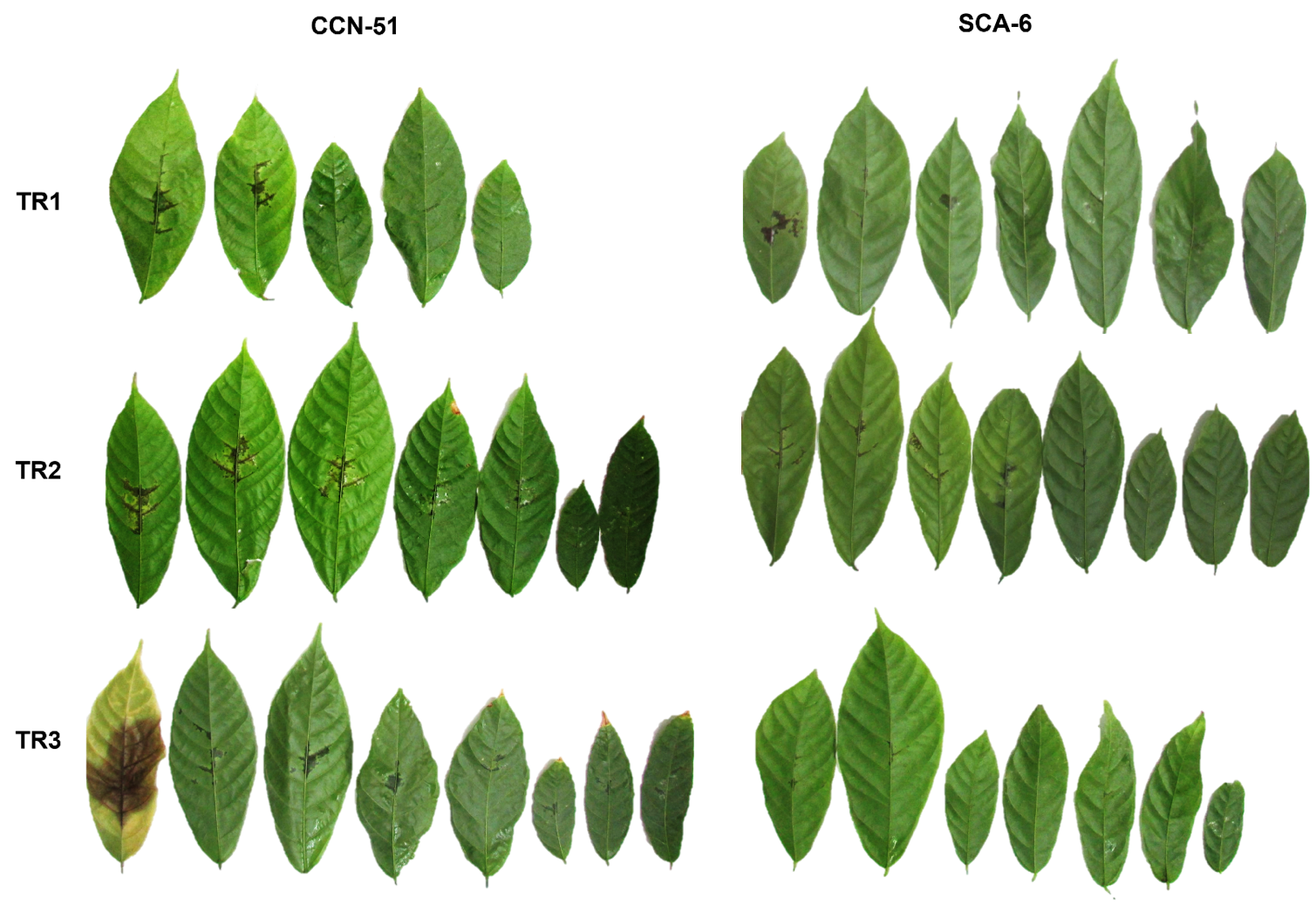


**Biological Replicate 1 – 96 hours post inoculation**


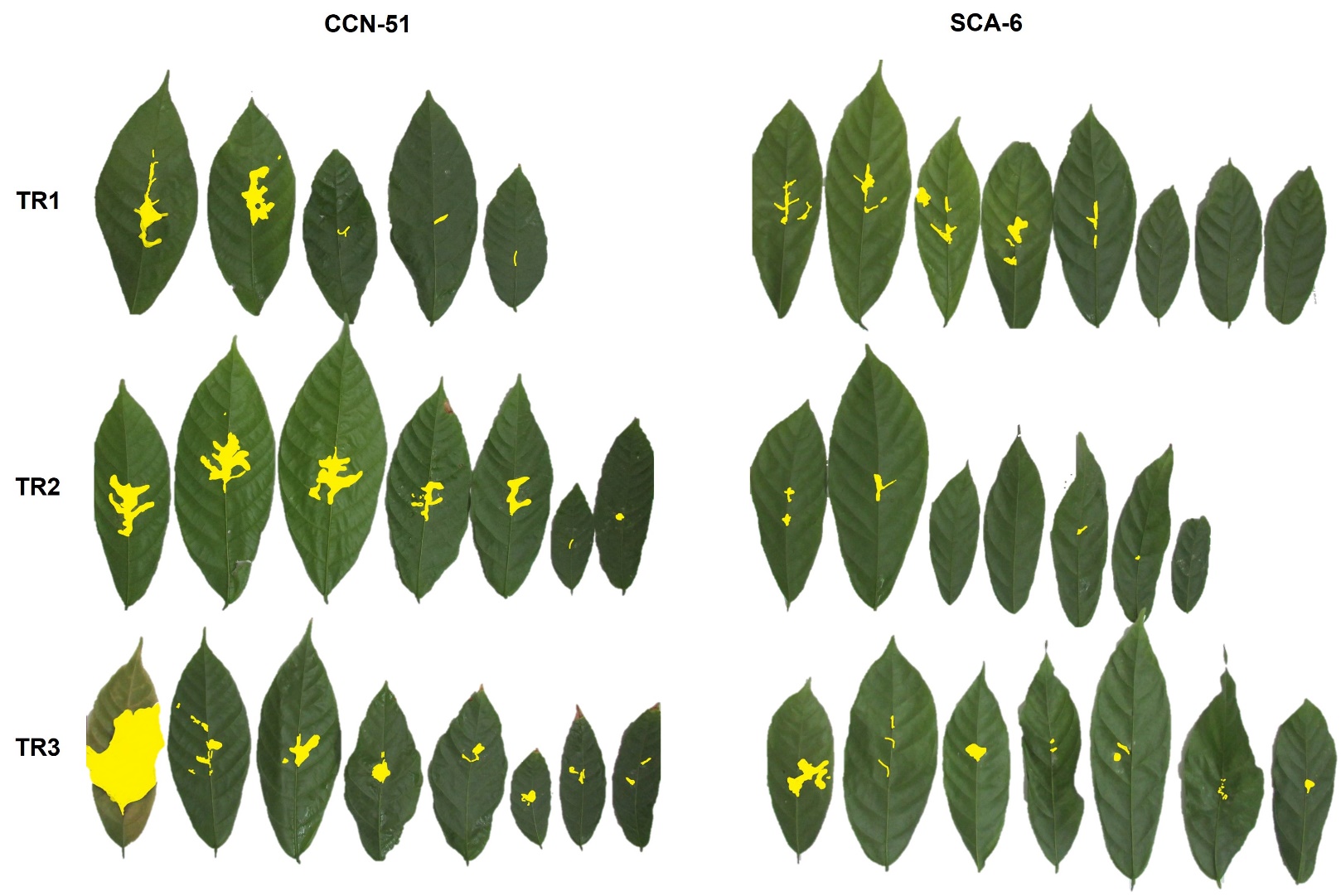


**Biological Replicate 2 - 96 hours post inoculation**


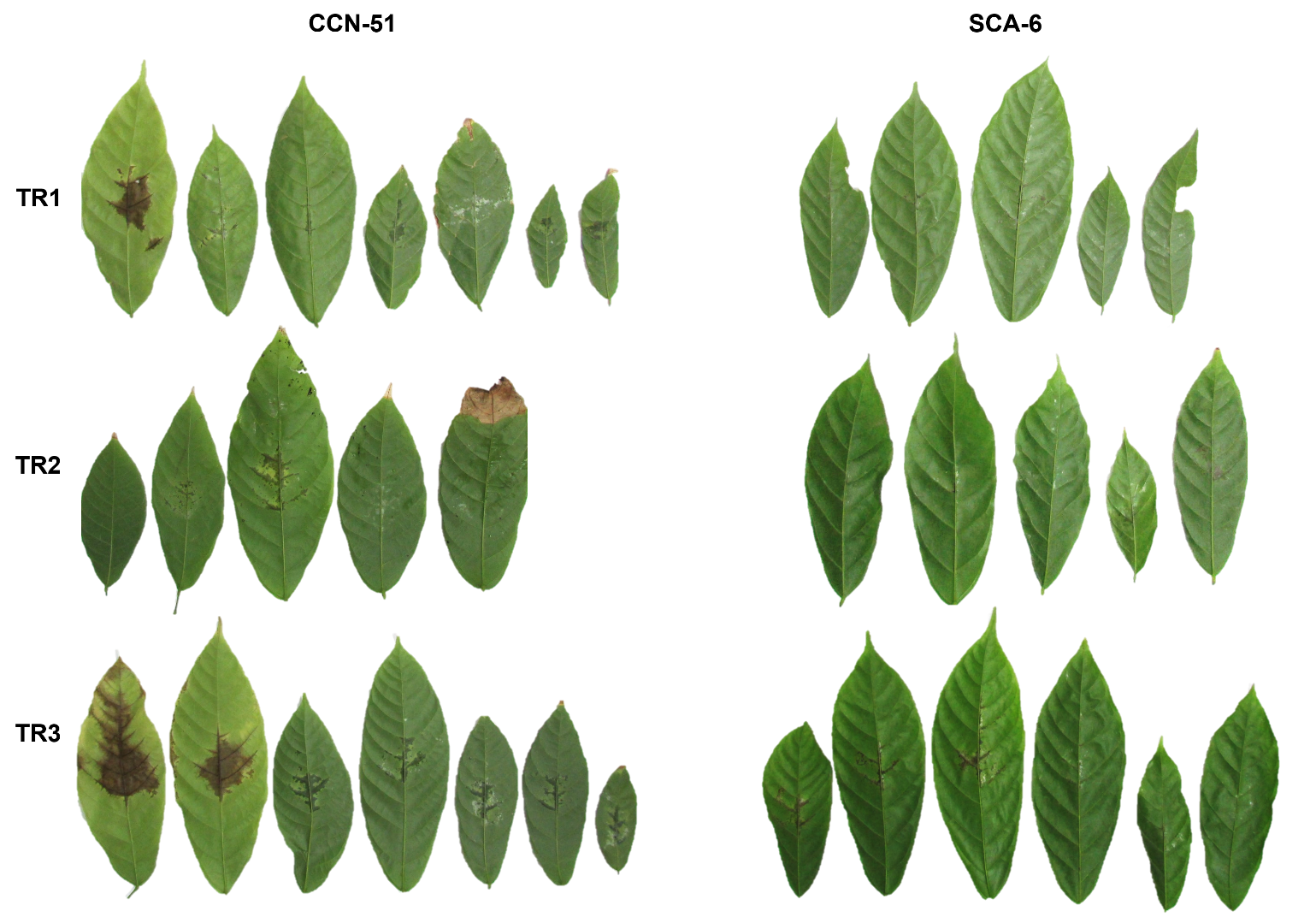


**Biological Replicate 2. 96 hours post inoculation**


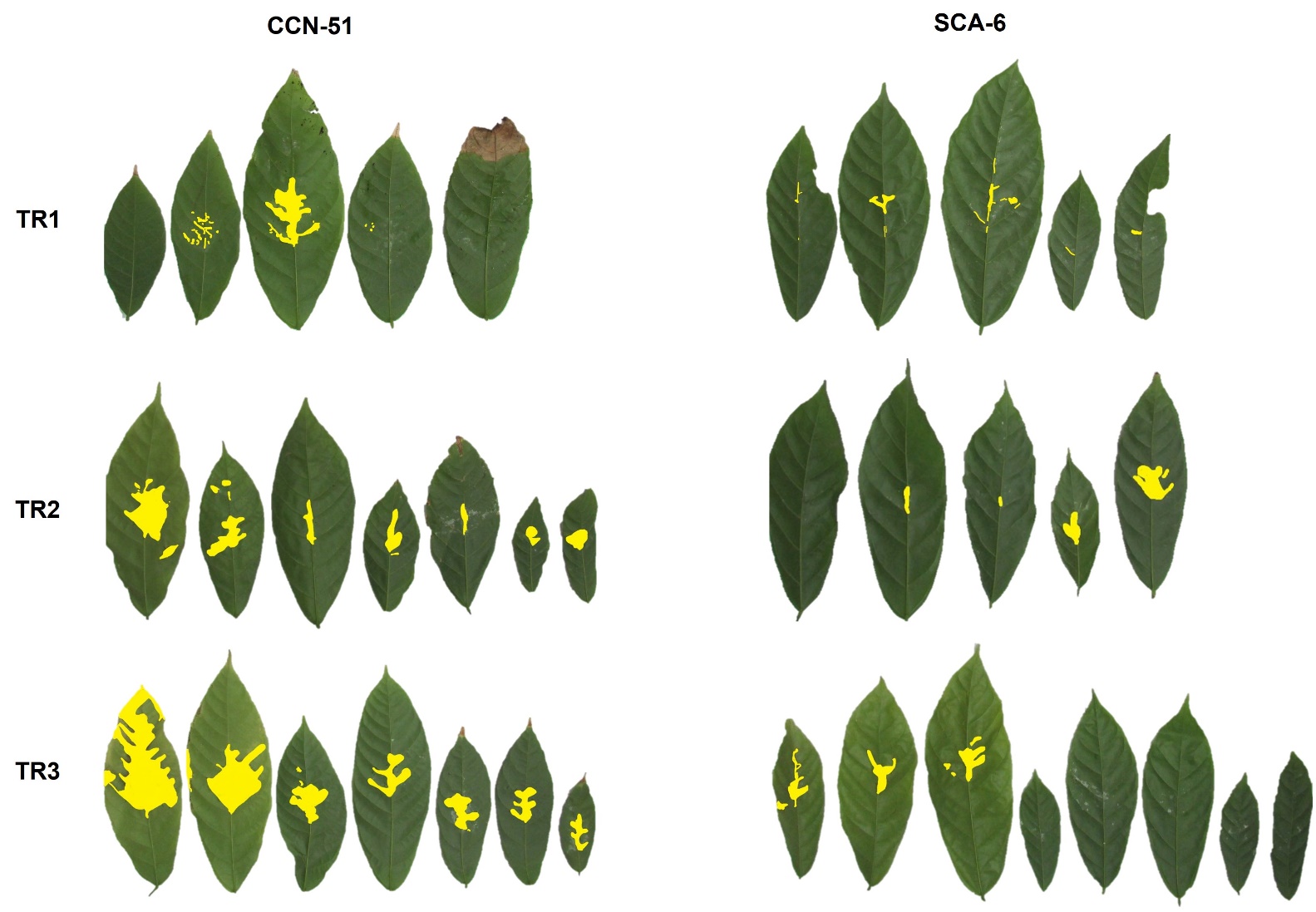


**Biological Replicate 3. 96 hours post inoculation**

**
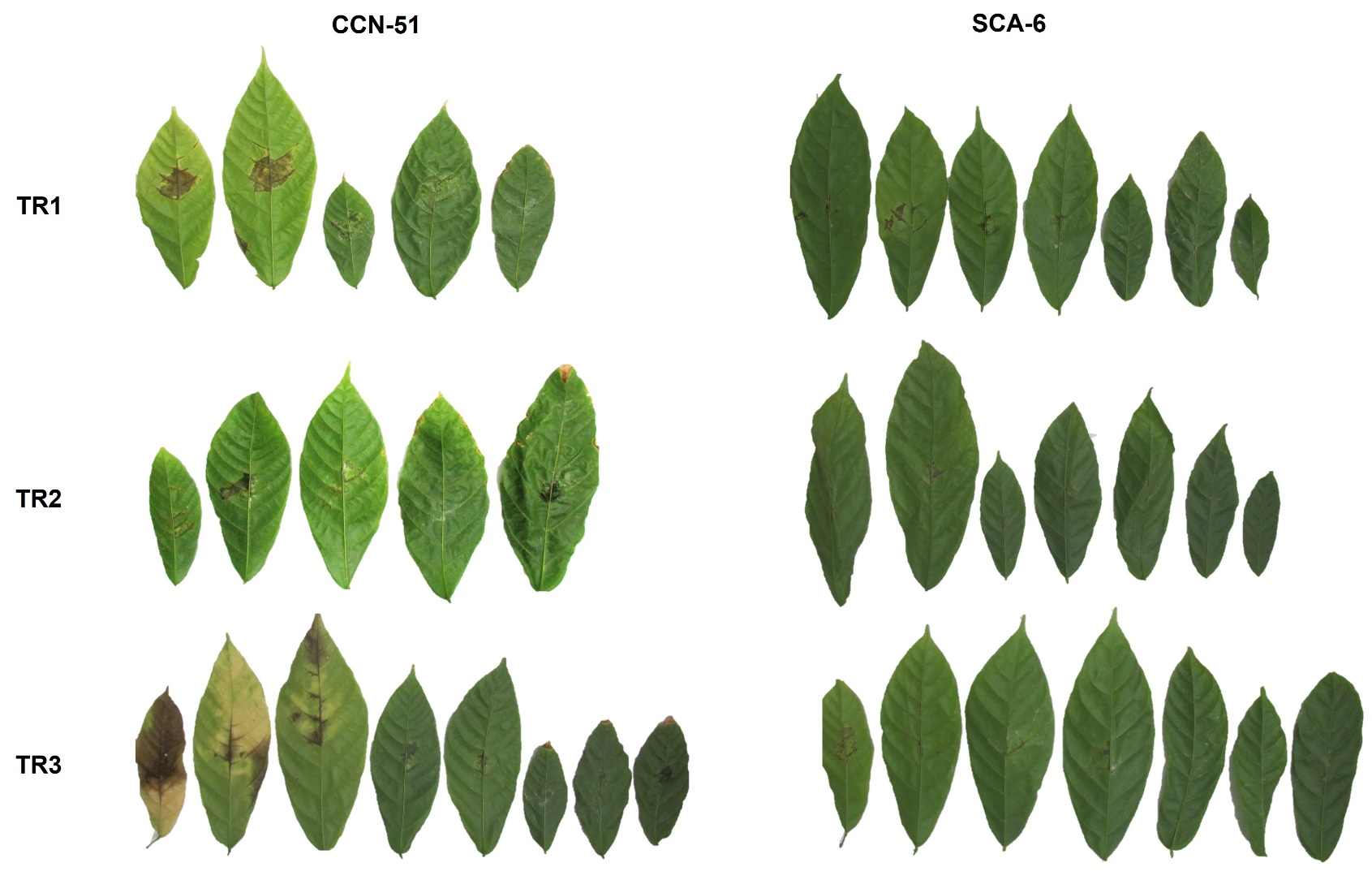
**

**Biological Replicate 3. 96 hours post inoculation**


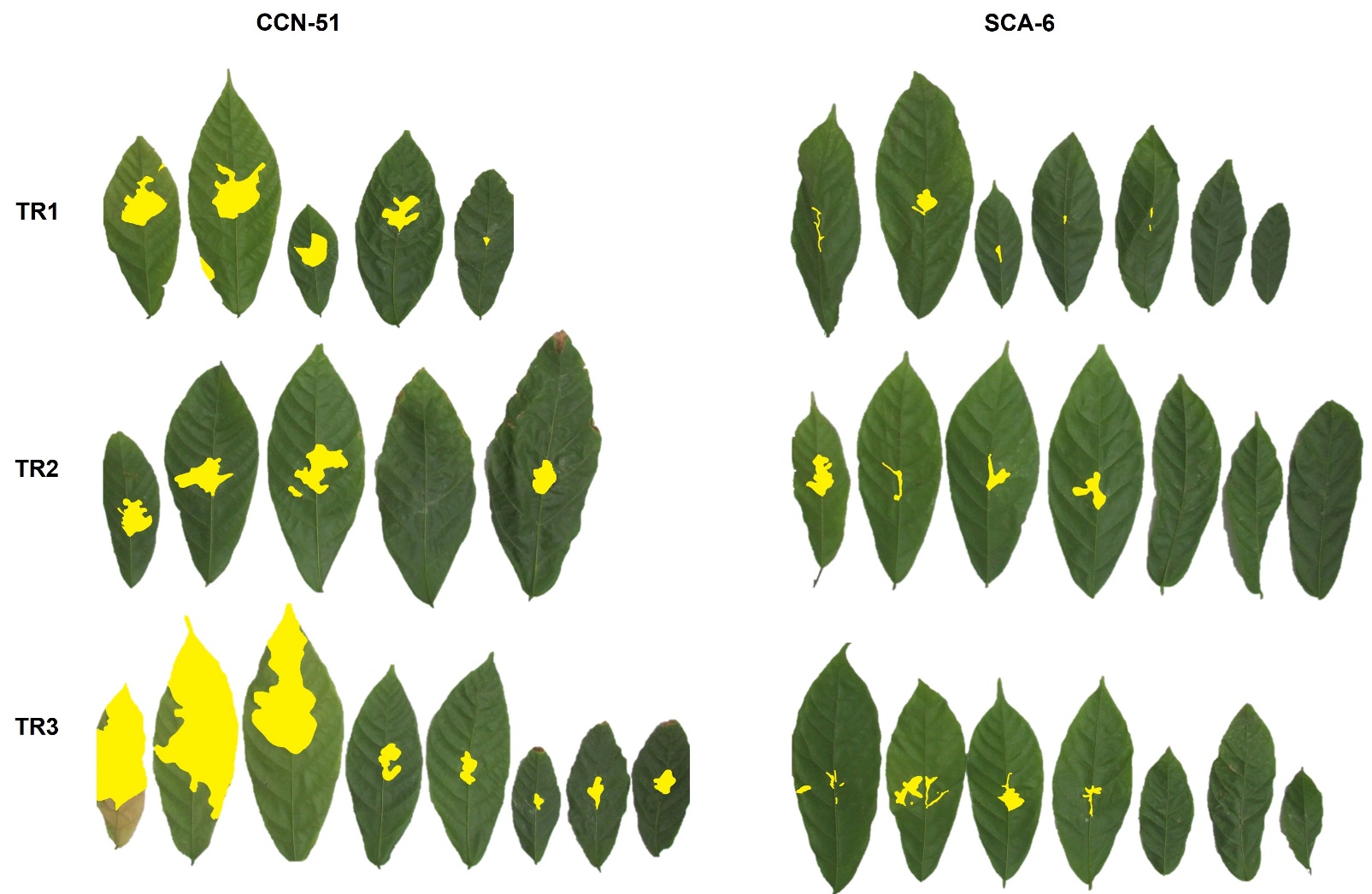

Supplement: Supplementary file 2 — Additional file 2. Images of the leaves from the two genotypes, CCN-51 and SCA-6, at 96 h after inoculation with Phytophthora palmivora. The lesion area caused by black pod was coloured in yellow to facilitate the recognition by the Compu Eye LSA software. CCN-51: susceptible genotype; SCA-6: tolerant genotype; TR: technical replicate (three plants per biological replicate). [file 13007_2020_656_MOESM2_ESM.docx]
