# Supplementary material for: A new method for the inoculation of Phytophthora palmivora (Butler) into cacao seedlings under greenhouse conditions
Source: Plant Methods. 2020 Aug 19;16:114. doi: 10.1186/s13007-020-00656-8 (PMC7437064; doi:10.1186/s13007-020-00656-8)
Supplement: Supplementary file 3 — Additional file 3. a. One-way ANOVA of leaf damage caused by P. palmivora. b. Tukey’s test for the average of the percentage of the lesion caused by P. palmivora. [file 13007_2020_656_MOESM3_ESM.docx]

**Additional file 3.**

1. One-way ANOVA of leaf damage caused by *P. palmivora*

| **Source of variation** | **Sum of squares** | **Degrees of freedom** | **Mean squares** | **F** | **p-value** |
| --- | --- | --- | --- | --- | --- |
| Genotype | 66.22 | 7 | 9.46 | 21.4 | <0.0001 |
| Error | 7.06 | 16 | 0.44 |  |  |
| Total | 73.28 | 23 |  |  |  |

1. Tukey’s test for the average of the percentage of the lesion caused by *P. palmivora.*

| **Genotype** | **Average** | **SD** | **Significance group** |
| --- | --- | --- | --- |
| CCN-51 T0 | 0.00 | 0 | A |
| SCA-6 T0 | 0.00 | 0 | A |
| CCN-51 T24 | 0.15 | 0.09 | A |
| SCA-6 T24 | 0.01 | 0.02 | A |
| CCN-51 T48 | 1.57 | 0.72 | A |
| SCA-6 T48 | 0.76 | 0.30 | A |
| CCN-51 T96 | 5.21 | 1.6 | B |
| SCA-6 T96 | 1.68 | 0.42 | A |

Different letters indicate significant differences at P < 0.05 according to the Tukey test. The experimental error was 0.38. SD: Standard deviation
